# Changes of Serum CTRP12 in Patients With Coronary Artery Disease After the Treatment of Percutaneous Coronary Intervention and Its Relationship With In‐Stent Restenosis

**DOI:** 10.1002/clc.70233

**Published:** 2026-01-12

**Authors:** Botao Zhao, Yuchao Shen

**Affiliations:** ^1^ Department of Cardiology Cangzhou Central Hospital Cangzhou Hebei China; ^2^ Department of Emergency Brain Academy District Cangzhou Central Hospital Cangzhou Hebei China

**Keywords:** C1q/tumor necrosis factor‐related protein, coronary artery disease, in‐stent restenosis, percutaneous coronary intervention, prediction

## Abstract

**Background:**

C1q/tumor necrosis factor‐related protein 12 (CTRP12) plays a protective role in coronary artery disease (CAD) by reducing vascular inflammation. Whether CTRP12 could predict the occurrence of CAD and in‐stent restenosis (ISR) occurrence after percutaneous coronary intervention (PCI) treatment remains unknown.

**Methods:**

This retrospective cohort study included patients with CAD who underwent PCI and had serum samples collected at baseline, 24 h, and 72 h post‐PCI. The Gensini score was used to assess the severity of coronary artery stenosis. Serum CTRP12 levels were measured using an ELISA kit. Follow‐up evaluation of ISR was performed using coronary angiography.

**Results:**

Serum CTRP12 levels were significantly lower in CAD patients than healthy controls (*p* < 0.001). A negative correlation was found between CTRP12 levels and both the Gensini score (r = −0.37, *p* < 0.001) and hs‐CRP levels (r = −0.43, *p* < 0.001). Dynamic analysis revealed a significant reduction in CTRP12 levels 24 h post‐PCI, with partial recovery observed at 72 h, although levels remained lower than baseline. Patients with ISR consistently demonstrated lower CTRP12 levels than those without ISR (NISR) at all time points. Receiver operating characteristic curve analysis indicated that CTRP12 levels at 24 h post‐PCI exhibited the highest predictive accuracy for ISR occurrence.

**Conclusion:**

Serum CTRP12 level may serve as a potential biomarker for diagnosing CAD and predicting ISR occurrence, with significant correlations to disease severity and dynamic changes after PCI.

## Summary

- What is known about the topic?
  ∘ C1q/tumor necrosis factor‐related protein 12 (CTRP12) plays a protective role in coronary artery disease (CAD) by reducing vascular inflammation.
- What′s new?
  ∘ Serum CTRP12 levels were significantly lower in CAD patients than healthy controls.
  ∘ Dynamic analysis revealed a significant reduction in CTRP12 levels 24 h post‐PCI.
  ∘ Serum CTRP12 level may serve as a potential biomarker for diagnosing CAD and predicting ISR occurrence.

## Graphical abstract

Serum CTRP12 levels are significantly lower in CAD patients, and significant correlate to disease severity and dynamic changes after PCI. They may serve as a potential biomarker for diagnosing CAD and predicting ISR occurrence.

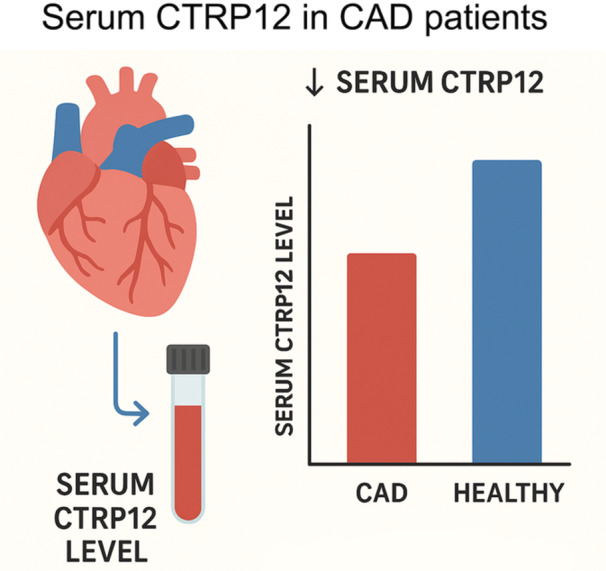

**Abbreviations**
CAD: coronary artery disease
CTRP12: C1q/tumor necrosis factor‐related protein 12
DALYs: Disability Adjusted Life Years
ISR: in‐stent restenosis
PCI: percutaneous coronary intervention

## 1 Introduction

Coronary Artery Disease (CAD) is a leading cause of mortality worldwide, significantly impacting global health and economies. It is characterized by the narrowing or blockage of coronary arteries, primarily due to atherosclerosis, which can lead to myocardial infarction and other serious cardiovascular events [1]. CAD is the foremost cause of mortality and loss of Disability Adjusted Life Years (DALYs) globally, with a significant burden in low and middle‐income countries. It accounts for nearly 7 million deaths and 129 million DALYs annually, posing a substantial economic burden worldwide [2]. CAD is influenced by several risk factors, including diabetes mellitus, hypertension, smoking, hyperlipidemia, obesity, and psychosocial stress [3]. Genetic studies have identified numerous loci associated with CAD risk, highlighting the heritable nature of the disease [4, 5]. The incidence of CAD also increases with age, and it is more prevalent in certain racial and ethnic groups, with South Asians having the highest rates [6].

Percutaneous coronary intervention (PCI) is a widely used procedure for treating CAD, particularly in complex coronary conditions [7]. PCI can significantly reduce the mortality rate of patients with coronary heart disease and improve their symptoms. However, in‐stent restenosis (ISR) occurrence after the procedure is a major challenge in PCI [8]. ISR is associated with an increased incidence of adverse cardiac events, including higher rates of myocardial infarction and target vessel revascularization, and is an important factor influencing the long‐term efficacy and prognosis following PCI [9]. The mechanisms underlying ISR remain unclear, as it is a complex pathological process involving multiple contributing factors. Some studies indicate that post‐PCI ISR is primarily localized to the site of vascular injury, where neointimal hyperplasia and de novo atherosclerosis led to lumen re‐narrowing, with inflammation serving as a key driver of ISR development [10].

Adipokines, bioactive molecules secreted by adipose tissue, play essential roles in the pathophysiology of cardiovascular diseases, including CAD [11]. Among these molecules, members of the C1q/tumor necrosis factor‐related protein (CTRP) superfamily have attracted increasing attention for their involvement in metabolic regulation and inflammatory signaling [12]. Adiponectin is one of the most extensively characterized CTRPs and has been shown to regulate obesity‐related metabolic disturbances while conferring protection against cardiovascular disorders [13, 14]. CTRP12 is a distinct paralog within the CTRP family, and accumulating evidence indicates that its biological actions, particularly in atherosclerosis, are mechanistically independent from those of adiponectin [11]. It was demonstrated that CTRP12 mitigated de novo atherosclerosis by enhancing reverse cholesterol transport efficiency and suppressing vascular inflammation via the miR‐155‐5p/LXRβ pathway, indicating that CTRP12 was associated with ISR occurrence in CAD after PCI treatment [11]. Notably, CTRP12 exhibits unique anti‐inflammatory and anti‐atherogenic activities compared with other CTRP family members, independently inhibiting macrophage activation, reducing vascular smooth muscle cell proliferation, and modulating vascular remodeling‐processes central to CAD progression and ISR [11, 15]. CTRP12 levels also respond dynamically to vascular injury and inflammatory stress, suggesting potential utility as a circulating biomarker in the peri‐procedural period of PCI‐treated patients [16]. Despite these insights, the specific contribution of CTRP12 to CAD pathogenesis and its role in post‐PCI complications, particularly ISR, remains insufficiently clarified [17].

Therefore, this study aims to explore the difference in serum CTRP12 levels between patients with CAD and healthy individuals and to investigate its relationship with the severity of coronary artery disease and high‐sensitivity C‐reactive protein (hs‐CRP) levels. Additionally, we aim to observe the dynamic changes of serum CTRP12 levels in CAD patients before and after PCI at 24 and 72 h. Furthermore, we seek to evaluate the predictive value of serum CTRP12 levels at different time points for ISR in patients.

## 2 Materials and Methods

### 2.1 Participants

This study is a retrospective cohort analysis of patients with CAD who underwent PCI at our institution. The study included patients with available serum samples collected before PCI, 24 h, and 72 h post‐PCI and who were successfully followed for 1 year after the procedure. The study was approved by the Ethics Committee of Cangzhou Central Hospital, and written informed consent was waived as this is a retrospective analysis.

### 2.2 Inclusion Criteria

Patients were included in the study if they met the following criteria: (1) A diagnosis of CAD according to the criteria outlined in the *Chinese Cardiovascular Disease Prevention Guidelines (2017)*, and underwent PCI; (2) Successful 1‐year follow‐up after PCI; (3) No history of valvular heart disease, cardiac mural thrombus, or heart failure; (4) Availability of serum samples collected before PCI, as well as 24 h and 72 h post‐PCI.

### 2.3 Exclusion Criteria

Patients were excluded from the study if they met any of the following criteria: (1) Presence of other cardiovascular diseases, such as dilated cardiomyopathy; (2) Development of vascular stenosis postoperatively due to causes such as arteritis; (3) History of coronary stent implantation or coronary artery bypass grafting; (4) Presence of severe diseases in other organs; (5) Missing clinical baseline data; (6) History of autoimmune diseases, hematologic disorders, or malignancies.

### 2.4 Gensini Score

All CAD patients underwent coronary angiography. The severity of coronary artery stenosis was assessed using the Gensini scoring system, which evaluates both the location and degree of the lesions. The scoring criteria for lesion location are as follows: small branches are assigned 0.5 points; the proximal, middle, and distal segments of the right coronary artery, distal segment of the left anterior descending artery (LAD), and the middle and distal segments of the left circumflex artery (LCX) are assigned 1.0 point; the mid‐segment of the LAD is assigned 1.5 points; the proximal segments of the LAD and LCX are assigned 2.5 points; and the left main coronary artery is assigned five points.

The degree of stenosis is graded as follows: 1%–25% stenosis is scored one point, 26%–50% stenosis is scored two points, 51%–75% stenosis is scored four points, 76%–90% stenosis is scored eight points, 91%–99% stenosis is scored 16 points, and 100% stenosis is scored 32 points. The total Gensini score is calculated as the product of the lesion location score and the degree of stenosis score. The patient′s overall Gensini score is the sum of the scores for all individual lesions. The Gensini score serves as a comprehensive index for assessing the severity of coronary artery stenosis in CAD patients.

### 2.5 Follow‐Up and Examination

Patients were followed up for 1‐year post‐PCI, during which they underwent coronary angiography at their discretion. ISR was defined as a stenosis greater than 50% within the stent lumen or a 5 mm segment at either end of the stent. The primary endpoint of the follow‐up was the occurrence of ISR or the results from the 1‐year follow‐up angiography.

### 2.6 Biomarker Measurement

CTRP12 levels in serum samples were measured using a commercial ELISA kit (CB13321‐Hu, COIBIO, Shanghai, China). According to the manufacturer′s specifications, the microplates provided with this kit are pre‐blocked during production with a blocking buffer containing bovine serum albumin or casein to minimize nonspecific binding. Serum samples were diluted at a 1:5 ratio by adding 40 μL of the kit‐supplied sample diluent followed by 10 μL of serum to each well, as recommended to ensure analyte concentrations fall within the assay′s dynamic range. Serum samples were incubated with a primary antibody for 20 min at room temperature and then overnight at 4°C. Following incubation, the plates were washed five times using the supplied wash buffer to remove unbound antibody. The wells received 50 µL of the secondary antibody and were incubated for 1 h at room temperature. After another wash, 50 µL of HRP‐conjugated enzyme was added to each well and incubated for 60 min at 37°C. Both the capture antibody (pre‐coated on the plate) and the HRP‐conjugated antibody are provided in ready‐to‐use form and do not require additional dilution, ensuring assay reproducibility. Following a final wash, 350 µL of substrate solution was applied, and the reaction was halted after 1 min by adding 50 µL of stop solution; the absorbance was measured at 450 nm within 5 min.

### 2.7 Statistical Analysis

Data comparisons between the two groups were performed using the Mann‐Whitney U test, unpaired *t*‐test with Welch′s correction, Fisher′s exact test, or Chi‐square test, as appropriate. Data are presented as n (percentage) or box plots. Statistical significance was determined if p‐values were less than 0.05.

## 3 Results

### 3.1 Demographic and Clinical Characteristics Between Healthy Control (HC) and CAD Patients

First, the demographic and clinical characteristics of 130 HC and 297 CAD patients included in the study were compared. There were no significant differences in age, gender, diabetes mellitus, hypertension, and smoking status between the two groups (Table 1), confirming that the control group is appropriately matched with the CAD patients in terms of baseline demographic and health characteristics.

**TABLE 1:** Demographic and clinical characteristic between healthy control (HC) and coronary artery disease (CAD) patients.

Characteristic	HC (*n* = 130)	CAD (*n* = 297)	*p* value
Age (years)			
< 60	37 (28.5%)	74 (24.9%)	0.473
≥ 60	93 (71.5%)	223 (75.1%)	
Gender (*n*, %)			
Male	76 (58.5%)	192 (64.6%)	0.233
Female	54 (41.5%)	105 (35.4%)	
Diabetes mellitus			
Yes	27 (20.8%)	79 (26.6%)	0.224
No	103 (79.2%)	218 (73.4%)	
Hypertension			
Yes	42 (32.3%)	111 (37.4%)	0.326
No	88 (67.7%)	186 (62.6%)	
Smoking			
Yes	53 (40.8%)	144 (48.5%)	0.170
No	77 (59.2%)	153 (51.5%)	

*Note:* The data are presented as n (percentage). The comparisons of data between the two groups were done by Fisher′s exact test.

### 3.2 The Serum CTRP12 Level Was Decreased in Patients With Coronary Artery Disease Compared to Healthy Control

**FIGURE 1:**
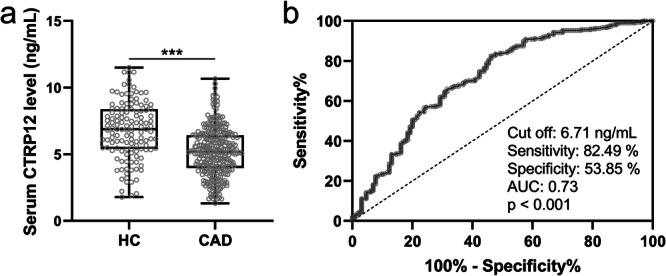
(a) Comparison of serum CTRP12 between healthy control (HC, *n* = 130) and patients with CAD (*n* = 297). Data were presented with box plot. ***p < 0.001 by Unpaired *t*‐test with Welch′s correction. (b) ROC analysis of the diagnostic value of serum CTRP12 for CAD.

Subsequently, the differences of serum CTRP12 levels between the two groups were analyzed. It was observed that serum CTRP12 levels were significantly reduced in CAD patients compared to HCs, with a p‐value less than 0.001 (Figure 1a). Receiver Operating Characteristic (ROC) curve analysis was conducted to evaluate the diagnostic value of serum CTRP12 for CAD. The optimal cut‐off value was 6.71 ng/mL, with a sensitivity of 82.49% and a specificity of 53.85% (Figure 1b). The Area Under the Curve (AUC) was 0.73, indicating moderate diagnostic accuracy (Figure 1b). These findings suggested that serum CTRP12 might be a potential biomarker for diagnosing CAD.

### 3.3 Serum CTRP12 Negatively Correlated With Gensini Score and hs‐CRP in CAD Patients

**FIGURE 2:**
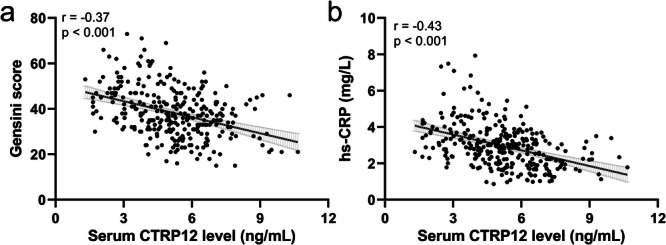
Pearson correlation analysis of serum CTRP12 with Gensini score (a) and hs‐CRP (b) in CAD patients (*n* = 297).

Previous findings confirmed a reduction of serum CTRP12 levels in patients with CAD. Subsequently, the relationships between CTRP12 levels and indicators of disease severity, including Gensini score and hs‐CRP, were investigated to determine whether CTRP12 levels correlate with the severity of CAD. Pearson correlation analysis revealed a negative correlation between serum CTRP12 levels and the Gensini score (r = −0.37, *p* < 0.001), indicating that lower CTRP12 levels are associated with more severe arterial stenosis (Figure 2a). A negative correlation between serum CTRP12 levels and hs‐CRP was also observed (Figure 2b, r = −0.43, *p* < 0.001), suggesting that reduced CTRP12 levels were associated with higher levels of inflammation as indicated by hs‐CRP. These results implied that serum CTRP12 levels might be a reflective biomarker of CAD severity.

### 3.4 Dynamic Changes of Serum CTRP12 in CAD Patients After PCI Treatment

**FIGURE 3:**
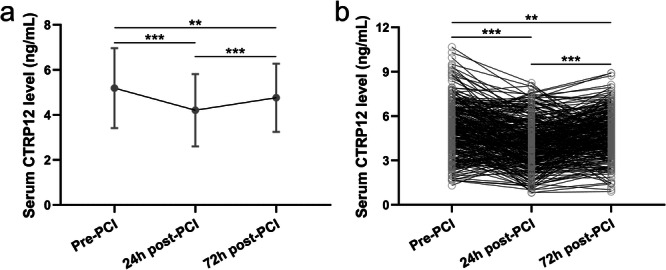
Changes of serum CTRP12 in CAD patients at the time of pre‐PCI, 24 h post‐PCI and 72 h post‐PCI (*n* = 297). Unpaired analysis (a) and paired analysis (b). **p < 0.01, ***p < 0.001 from Brown‐Forsythe ANOVA test following with Games‐Howell′s multiple comparisons test (a) and Repeated measures ANOVA summary following with Tukey′s multiple comparisons test (b).

To evaluate the acute vascular injury response and early inflammatory changes induced by PCI, we examined dynamic changes in circulating CTRP12 levels at 24 and 72 h following the procedure. Dynamic changes of serum CTRP12 levels were analyzed at three time points, including pre‐PCI, 24 h post‐PCI, and 72 h post‐PCI in patients with CAD. As shown in Figure 3a, a significant reduction of serum CTRP12 levels was observed 24 h post‐PCI compared to pre‐PCI levels. By 72 h post‐PCI, CTRP12 levels exhibited partial restoration, yet remained significantly lower than the levels observed pre‐PCI (Figure 3a). Paired analyses demonstrated that the mean serum CTRP12 levels were significantly reduced at 24 h post‐PCI, with a slight recovery observed by 72 h post‐PCI, although still significantly below the baseline levels (Figure 3b). These results suggested that serum CTRP12 might serve as a biomarker for monitoring the physiological responses of CAD patients to PCI.

### 3.5 Demographic and Clinical Characteristics Between NISR and ISR During 1 Year of Follow‐Up in CAD Patients Who Received PCI

ISR is a well‐known complication following PCI. Afterward, the CAD patients were classified into non‐in‐stent restenosis (NISR) and ISR groups according to the follow‐up. This study included 238 NISR patients and 59 ISR patients. The comparison showed no significant differences in factors such as age distribution, gender, diabetes, hypertension, smoking status, or coronary lesion location. However, the ISR group demonstrated significantly higher pre‐operative Gensini scores and hs‐CRP levels (Table 2, *p* < 0.001), suggesting a potential correlation with increased restenosis risk. Other metrics, including the number of stents, stent length, stent diameter, and LVEF, did not differ significantly (Table 2). These findings indicated that pre‐operative Gensini scores and hs‐CRP levels were correlated to the ISR occurrence.

**TABLE 2:** Demographic and clinical characteristic between non‐in‐stent restenosis (NISR) and in‐stent restenosis (ISR) during 1 year of follow‐up in coronary artery disease (CAD) patients received percutaneous coronary intervention (PCI).

Characteristic	NISR (*n* = 238)	ISR (*n* = 59)	*p* value
Age (years)			
< 60	64 (26.9%)	10 (16.9%)	0.132
≥ 60	174 (73.1%)	49 (83.1%)	
Gender (*n*, %)			
Male	156 (65.5%)	36 (61.0%)	0.545
Female	82 (34.5%)	23 (39.0%)	
Diabetes mellitus			
Yes	59 (24.8%)	20 (33.9%)	0.188
No	179 (75.2%)	39 (66.1%)	
Hypertension			
Yes	86 (36.1%)	25 (42.4%)	0.372
No	152 (63.9%)	34 (57.6%)	
Smoking			
Yes	111 (46.6%)	33 (55.9%)	0.245
No	127 (53.4%)	26 (44.1%)	
Coronary artery lesion location (*n*, %)			
Left main coronary artery	9 (3.8%)	5 (8.5%)	0.163
Left anterior descending	132 (55.5%)	35 (59.3%)	0.661
Left circumflex artery	115 (48.3%)	31 (52.5%)	0.565
Right coronary artery	80 (33.6%)	22 (37.3%)	0.647
Number of coronary artery lesions			
1	74 (31.1%)	19 (32.2%)	0.851
2	102 (42.9%)	23 (39.0%)	
≥ 3	62 (26.0%)	17 (28.8%)	
Number of stents	1.76 ± 0.65	1.87 ± 0.79	0.301
Stent length (mm)	37.24 ± 8.16	38.09 ± 8.31	0.226
Stent diameter (mm)	2.77 ± 0.74	2.68 ± 0.81	0.463
LVEF (%)	58.56 ± 8.09	56.92 ± 8.47	0.185
Gensini score before operation	36.96 ± 10.12	43.41 ± 12.04	< 0.001
hs‐CRP (mg/L)	2.77 ± 1.04	3.71 ± 1.47	< 0.001

*Note:* The data are presented as mean ± SD or n (percentage). The comparisons of data between the two groups were done by Mann Whitney test or Unpaired *t* test with Welch′s correction or Fisher′s exact test or Chi‐square test.

### 3.6 Serum CTRP12 Level Decreased in Patients With ISR at Different Time Points

**FIGURE 4:**
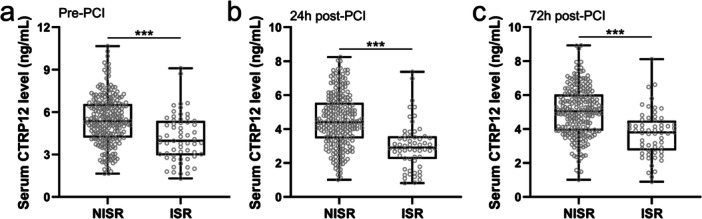
Comparisons of serum CTRP12 between NISR (*n* = 238) and ISR (*n* = 59) during one year follow‐up in CAD patients at the time of pre‐PCI (a), 24 h post‐PCI (b) and 72 h post‐PCI (c). Data were presented with box plot. ***p < 0.001 by Unpaired *t*‐test with Welch′s correction.

The differences of serum CTRP12 levels between the NISR and ISR groups were evaluated at three time points: pre‐PCI, 24 h post‐PCI, and 72 h post‐PCI. It was observed that serum CTRP12 levels were consistently and significantly lower in the ISR group compared to the NISR group at all three‐time points. The serum CTRP12 levels in the ISR group before PCI were significantly reduced compared to those in the NISR group (Figure 4a). Similarly, at 24 h post‐PCI, serum CTRP12 levels in the ISR group remained significantly lower than those in the NISR group (Figure 4b). At 72 h post‐PCI, serum CTRP12 levels in the ISR group were significantly reduced compared to the NISR group (Figure 4c). These findings indicated that lower serum CTRP12 levels were consistently associated with the ISR group, suggesting a potential association between reduced CTRP12 levels and the development of ISR.

To further exclude the potential confounding effect of diabetes on circulating CTRP12, subgroup analyses were conducted. After removing all individuals with diabetes, CTRP12 levels remained significantly lower in CAD patients (*n* = 218) than in healthy controls (*n* = 103) (Figure S1a, *p* < 0.001). In addition, diabetic CAD patients (*n* = 79) exhibited markedly lower CTRP12 levels compared with non‐diabetic CAD patients (*n* = 218) at the pre‐PCI, 24‐h, and 72‐h post‐PCI time points (Figure S1b–d). These findings indicate that both CAD and diabetes are associated with reduced circulating CTRP12, and the difference between healthy subjects and CAD patients is not explained by diabetes alone.

### 3.7 The Prediction Value of Serum CTRP12 at Three‐Time Points for ISR Occurrence After PCI

**FIGURE 5:**
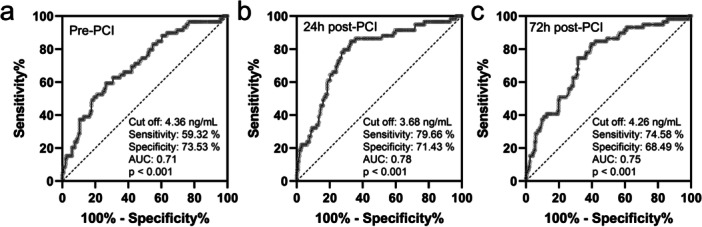
ROC analysis of serum CTRP12 at the time of pre‐PCI (a), 24 h post‐PCI (b) and 72 h post‐PCI (c) for prediction of in‐stent restenosis (*n* = 59) after one year follow‐up in CAD patients received PCI (*n* = 297).

The diagnostic potential of serum CTRP12 levels at different time points for predicting ISR was assessed through ROC curve analysis. At the pre‐PCI time, the AUC was 0.71, with a cut‐off value of 4.36 ng/mL, sensitivity of 59.32%, and specificity of 73.53% (Figure 5a). At 24 h post‐PCI, serum CTRP12 demonstrated the highest predictive accuracy, achieving an AUC of 0.78, with a cut‐off value of 3.68 ng/mL, sensitivity of 79.66%, and specificity of 71.43% (Figure 5b). At 72 h post‐PCI, the AUC was 0.75, with a cut‐off value of 4.26 ng/mL, sensitivity of 74.58%, and specificity of 68.49% (Figure 5c). These findings suggested that serum CTRP12 levels at 24 h post‐PCI offered the highest diagnostic accuracy for ISR, as evidenced by the greatest AUC and sensitivity. This highlights the potential utility of CTRP12 as a biomarker for the early detection of ISR.

## 4 Discussion

In this study, we aimed to evaluate the potential of CTRP12 as a biomarker for diagnosing CAD and predicting ISR after PCI. Our findings indicated that CTRP12 levels were significantly lower in CAD patients compared to healthy controls, and its dynamic changes post‐PCI correlated with disease severity and ISR occurrence.

In recent years, adipokines have attracted increasing attention for their role in atherosclerosis progression [18]. Adipose tissue, in addition to being an important energy storage site, has been recognized as the largest endocrine organ in the body, secreting various biologically active adipokines, such as leptin, adiponectin, TNF‐α, and retinol‐binding protein 4 (RBP4) [19]. These adipokines regulate energy balance, blood pressure, insulin sensitivity, and inflammatory responses and directly or indirectly contribute to the development of obesity‐related metabolic diseases [20]. The CTRP family plays a critical role in inflammation regulation [21]. As a representative member of the CTRP family, adiponectin enhances insulin sensitivity and improves insulin resistance. It also regulates endothelial cell function and affects vascular smooth muscle cell proliferation and migration, exerting protective effects in atherosclerosis development [22]. Clinical studies suggested that adipokines played a pivotal role in the pathogenesis of atherosclerosis and coronary artery disease [23, 24, 25].

CTRP12 is a member of the C1q/TNF‐related protein family, which shares functional similarities with adiponectin, a well‐known adipokine with anti‐inflammatory and anti‐atherogenic properties [26]. Serum levels of CTRP12 are independently associated with CAD and are correlated with several CAD risk factors, suggesting a possible link between CTRP12 and pathogenic mechanisms of atherosclerosis [27]. Consistent with previous research, our study found lower CTRP12 levels in CAD patients, suggesting that CTRP12 may have a protective role in cardiovascular health. Hayato et al. found that CTRP12 regulated pathological vascular remodeling by attenuating macrophage inflammation and VSMC proliferation through TGF‐βRII/Smad2 signaling [28]. This protective effect suggests that CTRP12 may be a potential therapeutic target for CAD. It was also reported that CTRP12 protected against hypoxia/re‐oxygenation‐induced cardiomyocyte injury by inhibiting apoptosis, oxidative stress, and inflammation through enhanced Nrf2 signaling [29]. Therefore, the mechanism of CTRP12 in promoting CAD will be explored in TGFb and Nrf2 signaling.

PCI restores coronary blood flow, significantly improving symptoms in patients with CAD, reducing the incidence of cardiovascular events, and enhancing cardiac function and quality of life [7]. Post‐procedural complications, including vascular injury and ISR, remain significant concerns in CAD patients receiving PCI. Therefore, exploring the dynamic changes of CTRP12 during PCI is crucial for understanding its involvement in the pathophysiology of ISR. Our study′s dynamic changes in serum CTRP12 levels post‐PCI reflect the physiological response to coronary intervention. CTRP12 levels were significantly reduced 24 h after PCI and showed partial recovery by 72 h, although they remained below baseline. These findings suggest that CTRP12 may be involved in the early inflammatory response following PCI, which is a critical period for assessing patient outcomes. Zhang et al. found that the peripheral venous serum CTRP12 levels in acute myocardial infarction patients were significantly decreased before PCI, continued to decrease on the 3rd postoperative day, and showed an increasing trend on the 5th and 7th postoperative days [30], which was different from our observation in CAD patients after PCI treatment. The marked reduction in CTRP12 at 24 h post‐PCI is possibly attributable to the acute inflammatory response and endothelial injury induced by balloon dilation and stent implantation. Because CTRP12 suppresses pro‐inflammatory cytokines such as IL‐6 and TNF‐α, its downregulation under acute inflammatory stress may explain the immediate postoperative decline. The partial recovery observed at 72 h may reflect the early resolution of vascular inflammation and the initiation of endothelial repair. Together, these temporal changes suggest that CTRP12 is highly responsive to PCI‐related vascular injury and may serve as an indicator of early postoperative inflammatory dynamics.

ISR remains a significant challenge following PCI, contributing to poor long‐term outcomes in CAD patients. In our study, serum CTRP12 levels were consistently lower in patients who developed ISR than those without ISR across all time points including pre‐PCI, 24 h post‐PCI, and 72 h post‐PCI. These findings supported the potential of CTRP12 as a predictive biomarker for ISR. ROC curve analysis further highlighted the diagnostic value of CTRP12, particularly 24 h post‐PCI, with a sensitivity of 79.66% and specificity of 71.43%. These results were in line with previous research suggesting that biomarkers reflecting endothelial dysfunction, vascular inflammation, and plaque formation can predict ISR occurrence [31]. The consistent decrease in CTRP12 levels in patients with ISR may indicate its role in exacerbating vascular inflammation and endothelial dysfunction, key contributors to ISR development. These findings suggested that CTRP12 could be used as a non‐invasive tool for early prediction of ISR, enabling clinicians to intervene earlier and improve long‐term outcomes.

Previous studies indicated that CTRP12 functioned anti‐inflammatory effects by suppressing cytokines such as IL‐6 and TNF‐α and by enhancing insulin sensitivity [32]. Because diabetes is marked by chronic low‐grade inflammation and impaired insulin signaling, the markedly lower CTRP12 levels observed in diabetic CAD patients may reflect a greater inflammatory and metabolic burden [27]. This suggests that reduced CTRP12 may serve both as a marker and a potential contributor to the heightened vascular inflammation that could increase ISR susceptibility in diabetic individuals. Further mechanistic studies are needed to clarify how CTRP12 interacts with inflammatory and metabolic pathways to influence restenosis risk in diabetes.

While our study presents promising findings, several limitations must be acknowledged. First, the retrospective design of the study limits the ability to establish causality between CTRP12 levels and the development of CAD or ISR. Prospective studies are required to confirm the temporal relationship between CTRP12 and disease progression. Second, the relatively small sample size, particularly for the ISR group, may have impacted the statistical power of the analysis. Larger, multicenter studies with diverse populations are needed to validate the predictive value of CTRP12 in different ethnic groups and clinical settings. Third, while we focused on serum CTRP12 levels, additional biomarkers and genetic factors that may contribute to CAD and ISR were not explored. Future studies should incorporate a broader range of biomarkers, including inflammatory cytokines, lipid profiles, and genetic predispositions, to improve the accuracy of ISR prediction.

## 5 Conclusions

This study found significantly lower serum CTRP12 levels in CAD patients than in healthy controls, suggesting its potential as a CAD biomarker. CTRP12 levels negatively correlated with disease severity markers, such as the Gensini score and hs‐CRP, indicating its role in reflecting CAD progression. Post‐PCI, CTRP12 levels decreased at 24 h, with partial recovery by 72 h, highlighting its potential to monitor PCI responses. Additionally, lower CTRP12 levels were associated with ISR, with the highest predictive accuracy at 24 h post‐PCI. These results suggest CTRP12 as a promising biomarker for both CAD diagnosis and early ISR prediction, aiding clinical decision‐making after PCI.

## Conflicts of Interest

The authors declare no conflicts of interest.

## Supporting information


**Figure S1:** a, Comparison of serum CTRP12 between healthy control without diabetes mellitus (HC, n = 103) and CAD patients without diabetes mellitus (CAD, n = 218). Comparisons of serum CTRP12 between CAD patients with (n = 79) or without (n = 218) diabetes mellitus at the time of pre‐PCI (b), 24 hours post‐PCI (c) and 72 hours post‐PCI (d). Data were presented with box plot. **p < 0.01, ***p < 0.001 by Unpaired t‐test with Welch's correction.

## Data Availability

Data are available upon reasonable request by contacting the corresponding author.
