# Supplementary material for: Changes of Serum CTRP12 in Patients With Coronary Artery Disease After the Treatment of Percutaneous Coronary Intervention and Its Relationship With In‐Stent Restenosis
Source: Clin Cardiol. 2026 Jan 12;49(1):e70233. doi: 10.1002/clc.70233 (PMC12794282; doi:10.1002/clc.70233)
Supplement: Supplementary file 1 — Figure S1: a, Comparison of serum CTRP12 between healthy control without diabetes mellitus (HC, n = 103) and CAD patients without diabetes mellitus (CAD, n = 218). Comparisons of serum CTRP12 between CAD patients with (n = 79) or without (n = 218) diabetes mellitus at the time of pre‐PCI (b), 24 hours post‐PCI (c) and 72 hours post‐PCI (d). Data were presented with box plot. **p < 0.01, ***p < 0.001 by Unpaired t‐test with Welch's correction. [file CLC-49-e70233-s001.docx]

Supplementary materials


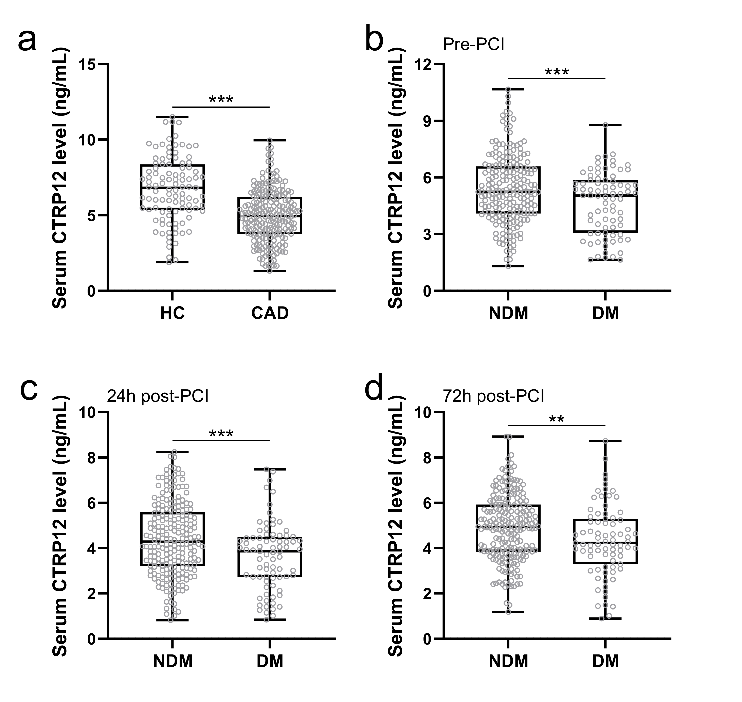


Figure S1. a, Comparison of serum CTRP12 between healthy control without diabetes mellitus (HC, n = 103) and CAD patients without diabetes mellitus (CAD, n = 218). Comparisons of serum CTRP12 between CAD patients with (n = 79) or without (n = 218) diabetes mellitus at the time of pre-PCI (b), 24 hours post-PCI (c) and 72 hours post-PCI (d). Data were presented with box plot. **p < 0.01, ***p < 0.001 by Unpaired t-test with Welch's correction.
